# Alternative Splicing of the Amelogenin Gene in a Caudate Amphibian, *Plethodon* *cinereus*

**DOI:** 10.1371/journal.pone.0068965

**Published:** 2013-06-26

**Authors:** Xinping Wang, Zeli Xing, Xichen Zhang, Lisai Zhu, Thomas G. H. Diekwisch

**Affiliations:** 1 College of Veterinary Medicine, Jilin University, Changchun, Jilin, China; 2 College of Dentistry, University of Illinois at Chicago, Chicago, Illinois, United States of America; University of Lausanne, Switzerland

## Abstract

As the major enamel matrix protein contributing to tooth development, amelogenin has been demonstrated to play a crucial role in tooth enamel formation. Previous studies have revealed amelogenin alternative splicing as a mechanism for amelogenin heterogeneous expression in mammals. While amelogenin and its splicing forms in mammalian vertebrates have been characterized, splicing variants of amelogenin gene still remains largely unknown in non-mammalian species. Here, using PCR and sequence analysis we discovered two novel amelogenin transcript variants in tooth organ extracts from a caudate amphibian, the salamander *Plethodon* *cinereus* . The one was shorter -S- (416 nucleotides including untranslated regions, 5 exons) and the other larger -L- (851 nt, 7 exons) than the previously published “normal” gene in this species -M- (812 nucleotides, 6 exons). This is the first report demonstrating the amelogenin alternative splicing in amphibian, revealing a unique exon 2b and two novel amelogenin gene transcripts in *Plethodon* *cinereus* .

## Introduction

Alternative splicing of the pre-mRNA is a powerful and versatile regulatory mechanism that allows the production of multiple protein variants from a single gene and affects the quantitative control of gene expression and the functional diversification of proteins [1,2]. As the major enamel matrix protein contributing to tooth development, amelogenin has been demonstrated to play a crucial role in tooth enamel formation [3–5]. Amelogenin has been identified in many species and its functions have been extensively studied in mammalian vertebrates, especially in mouse, rat, and bovine [3,6–23]. It has been reported that mammalian amelogenin exhibits heterogeneity in tooth organs and that different isoforms play different roles during enamel formation [7–9,11,13,14,24–27]. The heterogeneity of amelogenin is believed to be generated either from alternative splicing of amelogenin or posttranslational processing of the amelogenin protein [24,28–30]. The mammalian amelogenin gene is commonly organized into 7 exons (exon 1, 2, 3, 4, 5, 6, and 7) [31], except in rats and mice in which it contains two additional exons (8 and 9) [11,18]. Analysis of amelogenin gene genomic sequences revealed a piece of sequence homologous to exon 4 in mouse, rat, guinea pig, and deer mouse [31,32]. This short piece of sequence is located immediately upstream of exon 8 and designated as exon 4b [32]. However, exon 4b was never shown to be coding in any rodent species [31]. A full-length amphibian and sauropsids amelogenin gene was reported to contain 6 exons (1–3,5–7) [17,19–22], and the absence of exon 4 in lizard ( *Anolis* *carolinensis* ) and frog ( *Xenopus* *tropicalis* ) genomic DNA sequence has been confirmed [31]. However, a recent study has identified a novel amelogenin transcript in a reptile that contains 7 exons (1, 2, 3, 5, X, 6, 7). This transcript variant contains a unique exon X located between exon 5 and 6 [23].

Although the amelogenin gene and its splicing forms have been identified and characterized in mammals, little is known about amelogenin alternative splicing in non-mammalian vertebrates, especially in lipidosaurs and amphibians. To explore amelogenin alternative splicing in amphibians, we selected a caudate amphibian, the salamander *Plethodon* *cinereus* , as an animal model and employed PCR and sequence analysis to identify the potential amelogenin splicing transcripts. This study reports the discovery of two novel amelogenin splicing transcript variants and a unique exon 2b in *Plethodon* *cinereus* .

## Materials and Methods

### RNA isolation and RT-PCR

Three adult salamanders (6~8 cm) were chosen and euthanized according to the animal protocols specifically approved for this study by Jilin University Ethics Committee. RNA isolation was performed as previously reported [21]. Briefly, the salamander jaws were homogenized in TRI AGENT^®^ reagent (Sigma Co., St. Louis, Mo), then mixed with 0.2 volume of chloroform and shaken vigorously for 30 sec. After centrifugation at 12,000 x g for 20 min at 4 ^°^C, the aqueous phase was mixed with equal volume of isopropanol, centrifuged at 12,000 x g for 20 min at 4 ^°^C. The pellet was washed with 70% ethanol and dissolved in DEPC-treated H_2_O. The isolated RNA was kept at -80 ^°^C for further analysis.

The reverse transcriptase reaction was performed using SuperScript^TM^ II Reverse Transcriptase (Invitrogen, Carlsbad, CA). Briefly, cDNA was synthesized in a volume of 20 µl containing 25 mM Tris-HCI, pH 8.3, 37.5 mM KCI, 1.5 mM MgCI_2_, 5mM DTT, 0.25 mM each of dATP, dCTP, dGTP and dTTP, 40 units of RNase inhibitor, 200 units of M-MLV reverse transcriptase, 2 µg of total RNA, and 2.5 µM random primers. The cDNA synthesis was carried out at 42 ^°^C for 60 min. PCR amplification was done using Taq DNA polymerase (New England Biolabs, Ipswich, MA). The reaction was performed in a total volume of 50 µl containing 20 mM Tris-HCI, pH 8.4, 50 mM KCI, 3 mM MgCI_2_, 0.25 mM each of dATP, dCTP, dGTP and dTTP, 2.5 unit of Taq DNA polymerase, 1 µM of each primer, and 2 µl of the cDNA synthesized above. The amplification was done under the following conditions: denaturation at 94^°^C for 5 min; 35 cycles of 94^°^C for 30 sec, 56 ^°^C for 1 min, and 72^°^C for 1 min; and an additional extension at 72^°^C for 10 min. The primers were designed based on previously published sequence (Accession number: DQ069789.1) [33], and shown as follows: *P* *. cinereus* -sense 5’- CAAGGCCTTAACGAGACGGATACT -3’; *P* *. cinereus* -antisense 5’- ACTTTTCTCATTTGCGCTTCTTCA -3’.

### Cloning and sequencing

PCR products were purified and cloned to pGEM-T vector (Promega, Madison, WI). Recombinants were confirmed by digestion with EcoRI and further by sequencing at least three individual clones (Sangon Biotech, Shanghai). The resulting sequences were analyzed using DNAstar Lasergene software (DNASTAR, Inc, Madison, WI).

### 5’- and 3’-RACE 

5’- and 3’-RACE was performed to determine the 5’- and 3’-ends of the amelogenin gene sequence following a standard protocols, and the full-length of amelogenin gene sequence was reconstructed with 5’- and 3’-end sequences as previously described [23].

### Sequencing analysis

The nucleotide sequence served as a template for searching homologous sequences through GenBank (www.ncbi.nlm.nih.gov/genbank/). Alignment analysis of multiple sequences was performed using the Clustal W method [33]. The amelogenin amino acid sequence was deduced from the nucleotide sequence. The hydrophilicity-plots were generated using the Kyte and Doolittle algorithm [34]. The protein secondary structures were predicted using Psipred [35]. The protein homology detections and structure predictions were performed by HMM-HMM comparison using HHpred [36].

### Determination of exon 2b sequence

Based on the cDNA sequence of the *P* *. cinereus* amelogenin gene, two pair of primers was designed within the exons 2, 2b, 3 to determine the exon 2b sequence. The primer exon 2S/exon 3A was used to amplify the intron 2 fragment, and the primer pairs exon 2S/exon 2b-A and exon 2b-S/exon 3A were employed to amplify the potential fragments spanning the sequences from exon 2S to exon 2b, and from exon 2b to exon 3, respectively. The primer sequences are shown as follows. Exon 2S: 5’- GGACTTTGATTCTGCTCACTTGCC-3’; Exon 2b-A: 5’- CAGGCAGCGCTTCTGGTCCAG-3’; Exon 2b-S: 5’- CTGGACCAGAAGCGCTGCCTG-3’; Exon 3A: 5’- GATGTAACCTGGGTGGCTGGGATG-3’.

## Results

### Amelogenin gene amplification in *Plethodon* *cinereus* tooth organs

**Figure 1:**
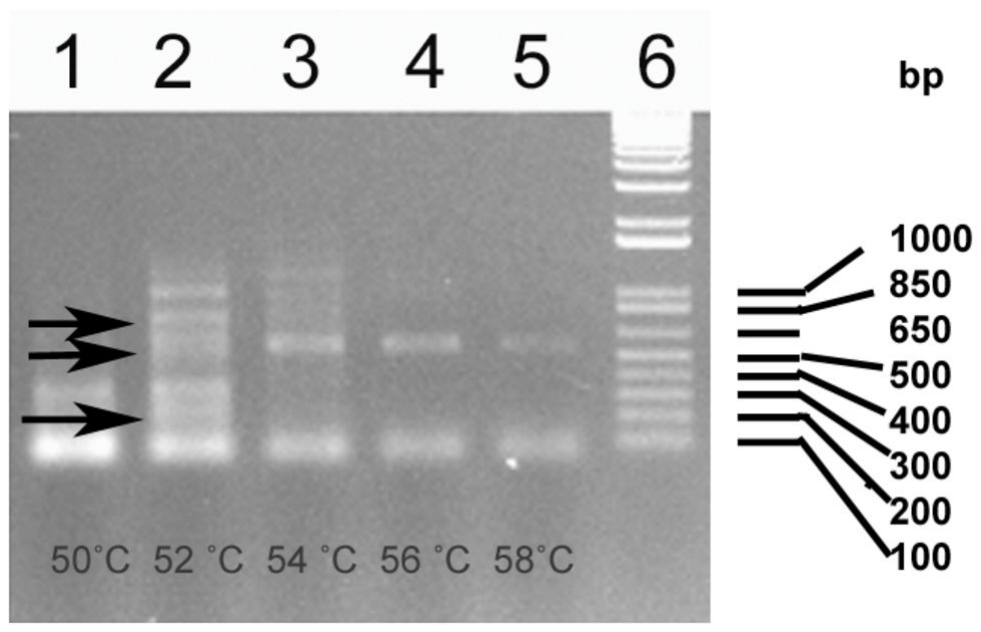
PCR amplification of potential amelogenin transcripts in *Plethodon* *cinereus* . The cDNA was synthesized from total RNA isolated from tooth organs of *Plethodon* *cinereus* and used to perform gradient PCR. PCR products amplified with different annealing temperatures were shown in lane 1(50°C), lane 2~3 (52°C, 54°C), lane 4 (56°C), and lane 5 (58°C), respectively. The arrow on the left showed the PCR products corresponding to AMEL-L, M and S (from top to bottom). Lane 6 is 100bp DNA plus ladder (Invitrogen).

To identify the potential amelogenin splicing transcripts in amphibians, the salamander *Plethodon* *cinereus* was chosen as an animal model and gradient PCR was employed to amplify the amelogenin gene. Several different amplified fragments were detected following electrophoresis, including two fragments with sizes 360 bp, and 560 bp, which were amplified with annealing temperatures at 50^°^C and 56^°^C ~58^°^C, respectively; while several other fragments were obtained at annealing temperature of 52^°^C ~54^°^C (Figure 1). Those results suggest the presence of potential amelogenin splicing forms in *P* *. cinereus* tooth organs.

**Figure 2:**
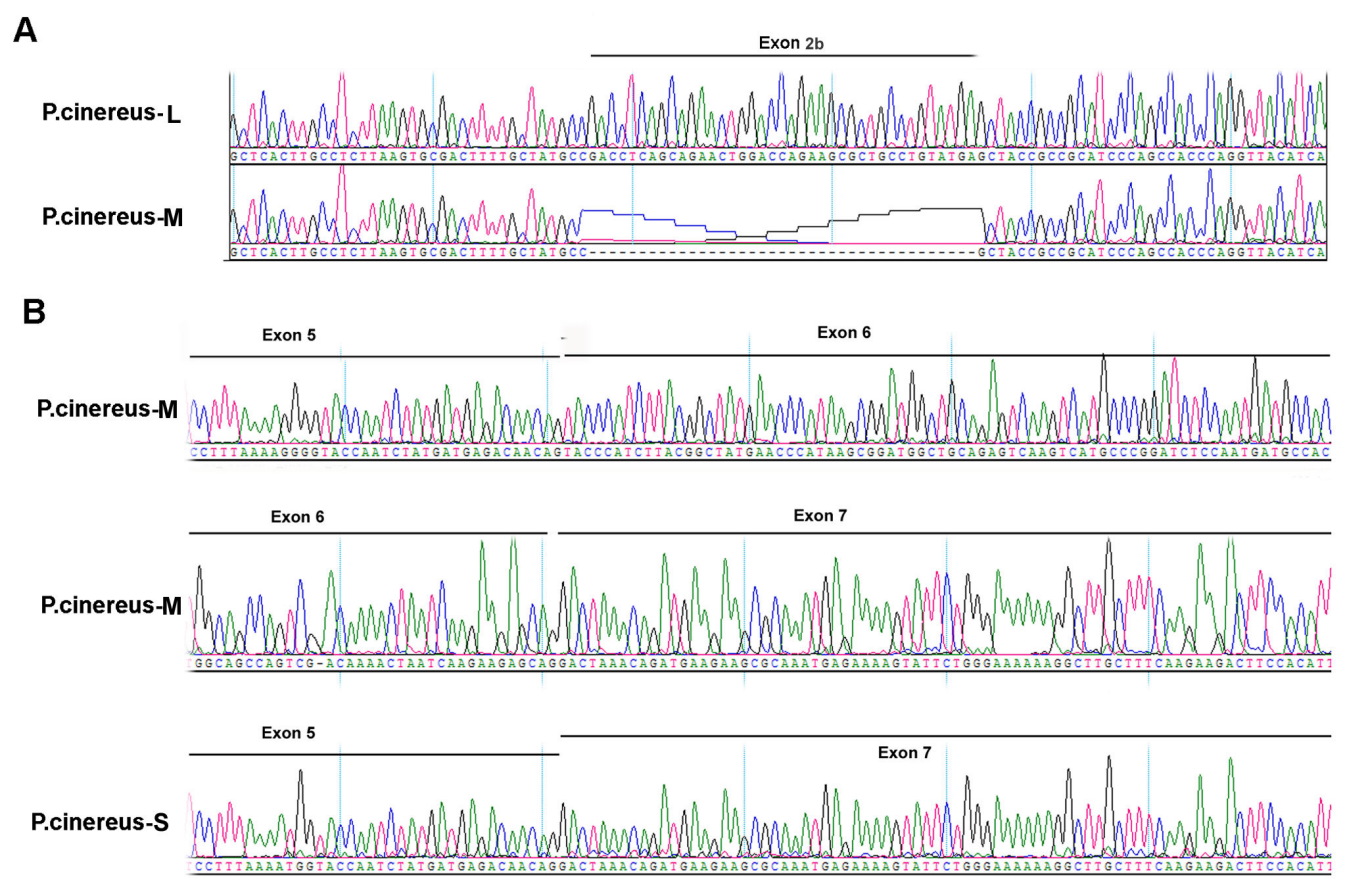
Identification of novel amelogenin gene splicing transcripts in *Plethodon* *cinereus* . (A) Chromatograms showed partial sequences of two *P* *. cinereus* amelogenin transcripts identified by sequencing PCR-amplified products. The cDNA clones corresponding to the transcripts were designated *as* *P* *. cinereus* -L (UP) and *P* *. cinereus* -M (low), respectively. In relation to *P* *. cinereus* -M, *P* *. cinereus* -L contains additional nucleotides (UP). (B) Chromatograms showed partial sequences of a short splicing form of the amelogenin transcript *P* *. cinereus* -S which is substantially shorter than *P* *. cinereus* *-M*. The upper and middle panels are partial discontinuous sequences of exon 6 in *P* *. cinereus* -M. The lower panel shows the partial sequence of *P* *. cinereus* -S. The exon region was labeled on the top of each chromatogram.

To determine potential splicing forms, PCR products were cloned into the pGEM-T vector and recombinants were identified by EcoRI enzyme digestion and confirmed by sequencing. After sequence analysis three clones with different sequence length were identified (Figure 2). These three clones were designated as *P* *. cinereus* -S (short), *P* *. cinereus* -M (medium), and *P* *. cinereus* -L (large), respectively.

### Identification of novel amelogenin gene splicing transcript variants in *P* *. cinereus* tooth organs

**Figure 3:**
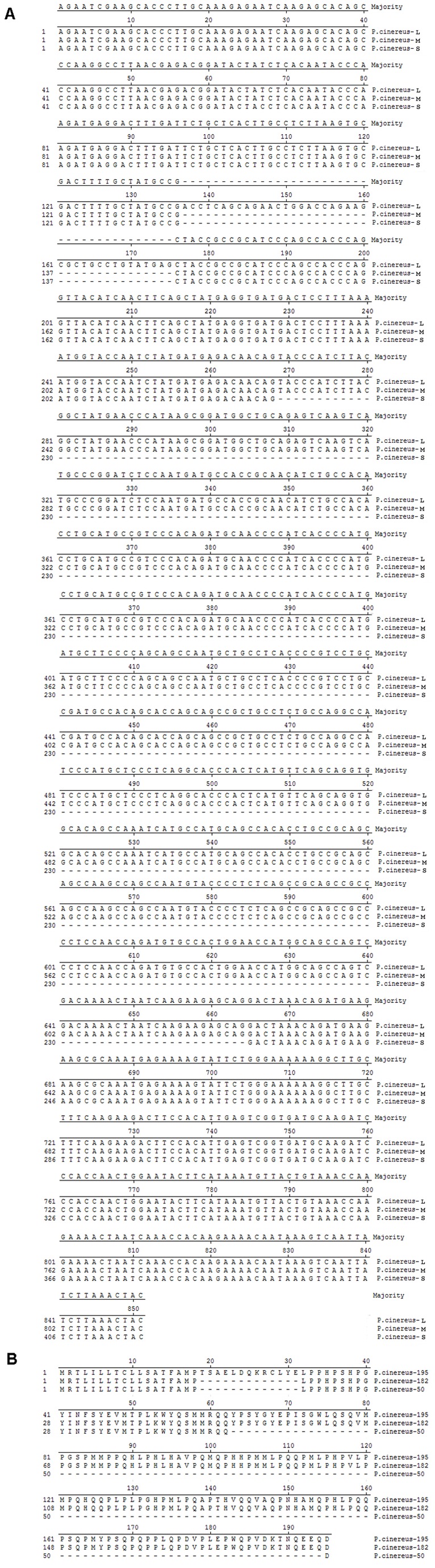
Novel amelogenin gene splicing forms in *Plethodon* *cinereus* . (A) Alignment analysis of the full-length salamander amelogenin gene cDNA sequence of *P* *. cinereus* -M with the novel salamander amelogenin cDNA sequence *P* *. cinereus* -L, and *P* *. cinereus* -S. The full-length of *P* *. cinereus* -M transcript is 812 bp, while the *P* *. cinereus* -L and *P* *. cinereus* -S transcripts are 851 bp and 416 bp, respectively. In relation to *P* *. cinereus* -M, the *P* *. cinereus* -L contains an additional 39 bp located between nucleotide 136 and 137, and *P* *. cinereus* -S is short of 396 nucleotides between nucleotides 230 and 629. The 5’-untranslated region contains 82 nucleotides upstream of the translation start codon ATG. The 3’-untranslated region contains 181 nucleotides downstream stop codon TAA. (B) Sequence analysis of the deduced amino acid sequence of *P* *. cinereus* -M, *P* *. cinereus* -L, and *P* *. cinereus* -S. In relation to *P*.*cinereus*-182 encoded by *P* *. cinereus* -M, the *P*.*cinereus*-195 encoded by *P* *. cinereus* -L contains an additional 13 amino acid residues located between amino acid residue 18 and 19. *P*.*cinereus*-50 was short of 123 amino acid residues between AA 49 and 181.

To characterize salamander amelogenin transcripts, the 5’- and 3’-RACE was used to obtain amelogenin cDNA sequences as described previously [23]. Sequence results demonstrated that full-length *P* *. cinereus* -L transcript contained 851 nucleotides, encoding 195 amino acid residues (GenBank accession number JX983173); *P* *. cinereus* -M transcript contained 812 nucleotides, encoding 182 amino acids (GenBank accession number DQ069790); and a short transcript *P* *. cinereus* -S consisted of 416 nucleotides, encoding 50 amino acid residues (GenBank accession number JX983174). The three amelogenin splicing transcripts had the same 5’-untranslated regions of 82 nucleotides upstream ATG translation start site, and 3’-untranslated region of 181 nucleotides downstream TAA stop codon (Figure 3A). The amelogenin proteins deduced from *P* *. cinereus* -M, *P* *. cinereus* -L and *P* *. cinereus* -S transcripts were designated as *P*.*cinereus*-182, *P*.*cinereus*-195, and *P*.*cinereus*-50, respectively (Figure 3B).

### Unique exon 2b in *P* *. cinereus* -L transcript of amelogenin gene

**Figure 4:**
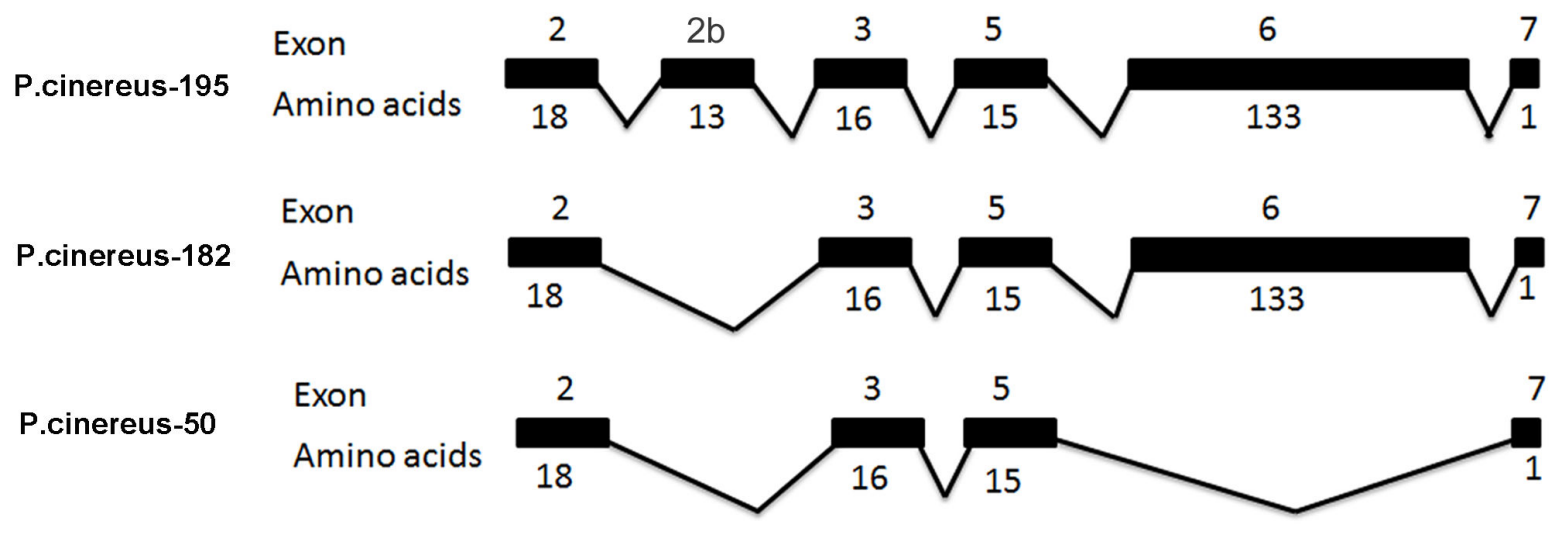
Novel salamander amelogenin gene cDNA structure. Analysis of novel salamander amelogenin sequence revealed the full-length amelogenin cDNA *P* *. cinereus* -L containing 7 exons including exon 1, 2, 2b, 3, 5, 6, and 7 (exon numbers is relative to published mammalian amelogenin exon numbers). Different from amelogenin genes published so far, a unique exon named exon 2b between exon 2 and exon 3 was detected. No corresponding sequence elements were detected resembling exon 4, suggesting that amelogenin exon 4 was skipped or deleted in *P* *. cinereus* . Unlike a previously reported full-length of *P* *. cinereus* -M isoform consisted of 6 exons, the novel splicing isoform of the *P* *. cinereus* -S transcript contains 5 exons including exon 1, 2, 3, 5, and 7.

To characterize the above transcripts, we further performed a sequence comparison of *P* *. cinereus* -M with *P* *. cinereus* -L. As shown in Figure 3A, P *. cinereous*-L contained an extra 39 nucleotides in relation to *P* *. cinereus* -M. The 39 nucleotides are located between nucleotide 136 and nucleotide 137 of the *P* *. cinereus* -M transcript, encoding 13 amino acid residues. Analysis of amelogenin gene organization of *P*.*cinereus*-195 revealed that the 13 amino acid residues do not match any known amelogenin exon sequence in GenBank. Compared to known amelogenin gene structures, the 13 amino acid residues are located between exon 2 and 3; these comprise a novel exon and are thus named exon 2b (Figure 3B). As shown in Figure 4, the full-length *P* *. cinereus* -L amelogenin gene consists of 7 exons including exons 1, 2, 2b, 3, 5, 6, and 7.

Since exon 2b was found to be a unique exon in amelogenin gene, we utilized its nucleotide and deduced amino acid sequences to search for the potential matching or homologous nucleotide/amino acid sequences in GenBank. Analysis did not find any exact matching nucleotide sequence, however amino acid sequence of exon 2b (TSAELDQKRCLYE) had a high homology identity with partial sequence of several genes (not shown).

### Unique amelogenin splicing form *P* *. cinereus* -S with a complete exon 6 sequence spliced out

To further define the sequence of *P* *. cinereus* -S transcript, the *P* *. cinereus* -S nucleotide sequence was aligned against *P* *. cinereus* -M. As shown in Figure 3A, 396 nucleotides of *P* *. cinereus* -M were spliced out when compared to *P* *. cinereus* -S; the deleted 396 nucleotides were located between nucleotide 230 and nucleotide 626 of the *P* *. cinereus* -M transcript. Further analysis identified *P*.*cinereus*-50 as a novel transcript variant consisting of 5 exons (1, 2, 3, 5, and 7) in which exon 6 was entirely spliced out (Figures 3, 4).

### Analysis of the origin of exon 2b

In order to determine whether the new sequences belongs to either the 3’ region of exon 2, or the 5’ region of exon 3, or more probably is located in the intronic region between exons 2 and 3, and therefore a new exon, PCR analysis was performed using primers exon 2S/exon 3A designed in these two exons to amplify the intron 2 sequences. Unfortunately, we failed to amplify the intron 2 fragment. Primer pairs exon 2S/exon 2b-A and exon 2b-S/exon 3 were also employed in an attempt to amplify the fragments between exon 2 and exon 2b, and between exon 2b and exon 3, however, no expected fragments were amplified. In absence of a complete salamander genomic sequence, we further performed an alignment analysis of exon 2b (39 nt) with the corresponding amelogenin intron 2 sequences in several species including human (GRCh37), mouse (NC_000086), green anole (NW_003339249), and Western clawed frog (NW_003163397.1). Alignment analysis did not identify a sequence with a typical exon/intron boundary character; however, it revealed a small sequence region in the intron 2 with moderate sequence identity to exon 2b (not shown). Those results suggest that exon 2b might be originated from the salamander amelogenin intron 2 sequences.

Since the paucity of genome sequence in non-mammalian vertebrates, especially in the *P* *. cinereus* , we further performed alignment analysis to test our hypothesis that exon 2b could also be the partial 3’ region of exon 2 or the 5’ region of exon 3 using the exon sequence named exon 2-2b (exon 2 + exon 2b) and exon 2b-3 (exon 2b + exon 3) to search the potential homologous sequence against the *Xenopus* amelogenin genomic sequences spanning the region from exon 2 to exon 3. *Xenopus* amelogenin genomic sequence is one of the few known amphibian amelogenin sequence. After alignment analysis, it did not show any sequence similarity of exon 2b to either 5’ region or 3’ region of intron 2, suggesting that exon 2b did not belong to a partial 3’ region of exon 2 or 5’ region of exon 3.

### Effect of Exon 2b sequence on the putative *P*.*cinereus*-195 protein structures

**Figure 5:**
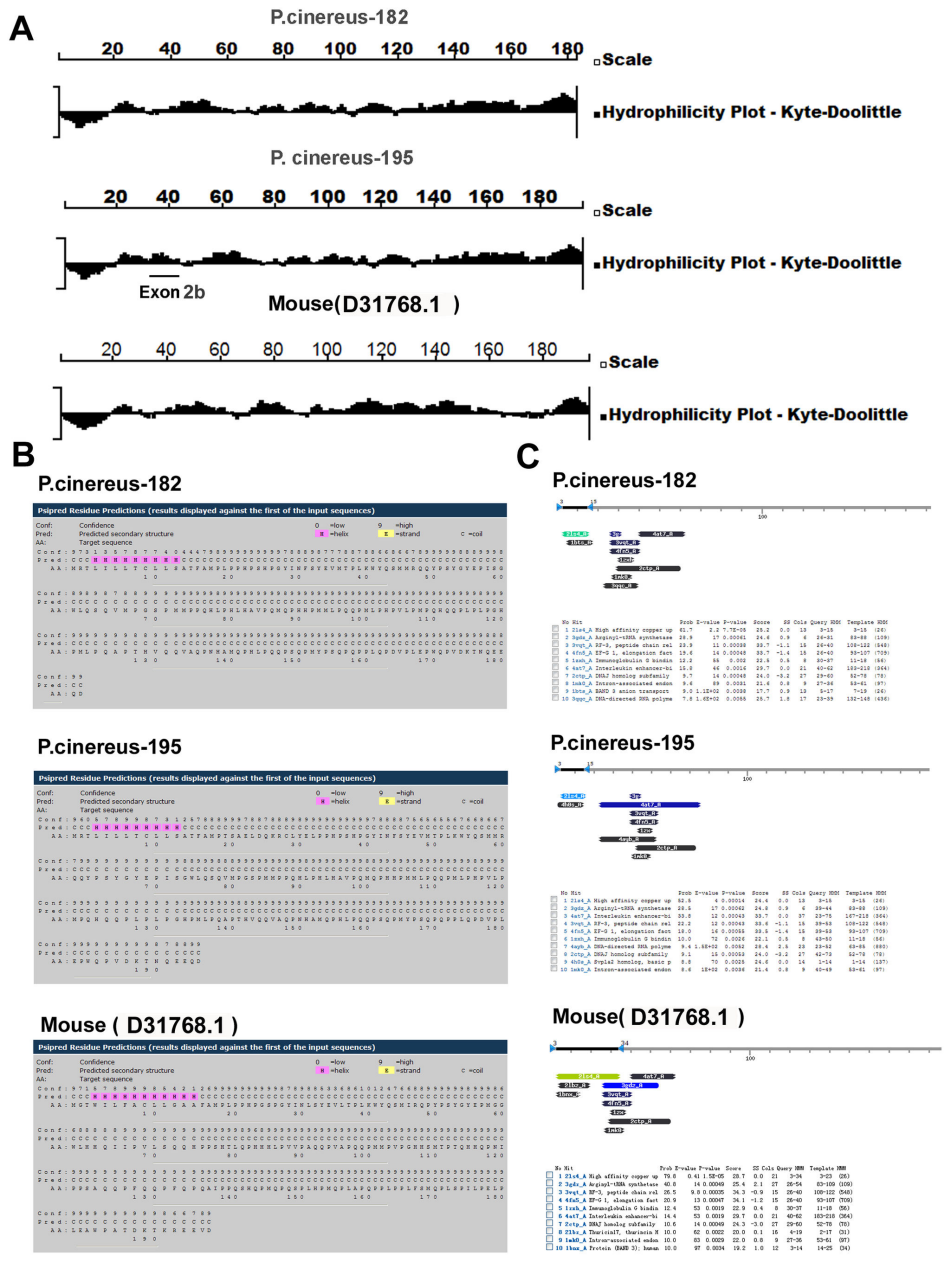
Effect of exon 2b sequence on the amelogenin protein structures. (A) Hydrophilicity-plot analysis using the Kyte and Doolittle algorithm. Hydrophilicity-plots of *P*.*cinereus*-182, *P*.*cinereus*-195 and mouse (D31768.1) were generated and compared. In relation to *P*.*cinereus*-182 and mouse amelogenin, the region underlined with a black line (middle panel) is the exon 2b sequence, which was hydrophilic. (B) Exon 2b has no significant effect on the secondary structure of P.cinereus-195 predicted by Psipred. Similar to that of mouse amelogenin (AA 4~12), one potential helical region for *P*.*cinereus*-182 (AA 4~11) and *P*.*cinereus*-195 (AA 4~11) was revealed by Psipred prediction. (C) Exon 2b sequence effects on the tertiary structures of *P*.*cinereus*-195. *P*.*cinereus*-182 and *P*.*cinereus*-195 were used as query sequence for homology detection and structure prediction by HMM-HMM comparison using HHpred. A bar graph summarizes the positions and color-coded significances of the database matches with the probability. A tabular hit lists probabilities, E-values, scores, and match regions in queries and templates.

To determine whether exon 2b has any effect on the amelogenin protein structure, the hydrophilicity-plots of *P*.*cinereus*-195 and *P*.*cinereus*-182 were generated using the Kyte and Doolittle algorithm [34], and secondary structures of putative *P*.*cinereus*-195, *P*.*cinereus*-182, and *P*.*cinereus*-50 were predicted using Psipred [35,36]. When compared to *P*.*cinereus*-182 and mouse amelogenin (D31768.1), exon 2b (AA 19-32, TSAELDQKRCLYE) of *P*.*cinereus*-195 turned out to be hydrophilic, which affected its N-terminal hydrophilicity-plot (Figure 5A). Psipred prediction revealed one potential helical region for *P*.*cinereus*-195 (AA 4~11), which is similar to the potential helix regions observed in *P*.*cinereus*-182 (AA 4~11) and mouse amelogenin gene (AA 4~12) (Figure 5B). Those results suggest that exon 2b has no significant effect on its secondary structure.

To further explore the potential effects of exon 2b sequence on the tertiary structures of amphibian amelogenins, *P*.*cinereus*-195, *P*.*cinereus*-182 and *P*.*cinereus*-195 were used as query sequences for protein homology detection and structure prediction by HMM-HMM comparison using HHpred. As shown in Figure 5C, representing proteins with closest homologs of structure or domains with *P*.*cinereus*-182 (Upper) or *P*.*cinereus*-195 (Middle) and mouse amelogenin gene (D31768.1) were listed. Although the majority of listed homologs/domains with P.cinereus-195 were similar to those of P.cinereus-182 and mouse amelogenin (D31768.1), there was still a unique homolog/domain which lacked in P.cinereus-182 and mouse amelogenin (Figure 5C), indicating that exon 2b sequence had an effect on its tertiary structure.

### Effect of splicing related exon 6 removal in *P*.*cinereus*-50 on protein structures

**Figure 6:**
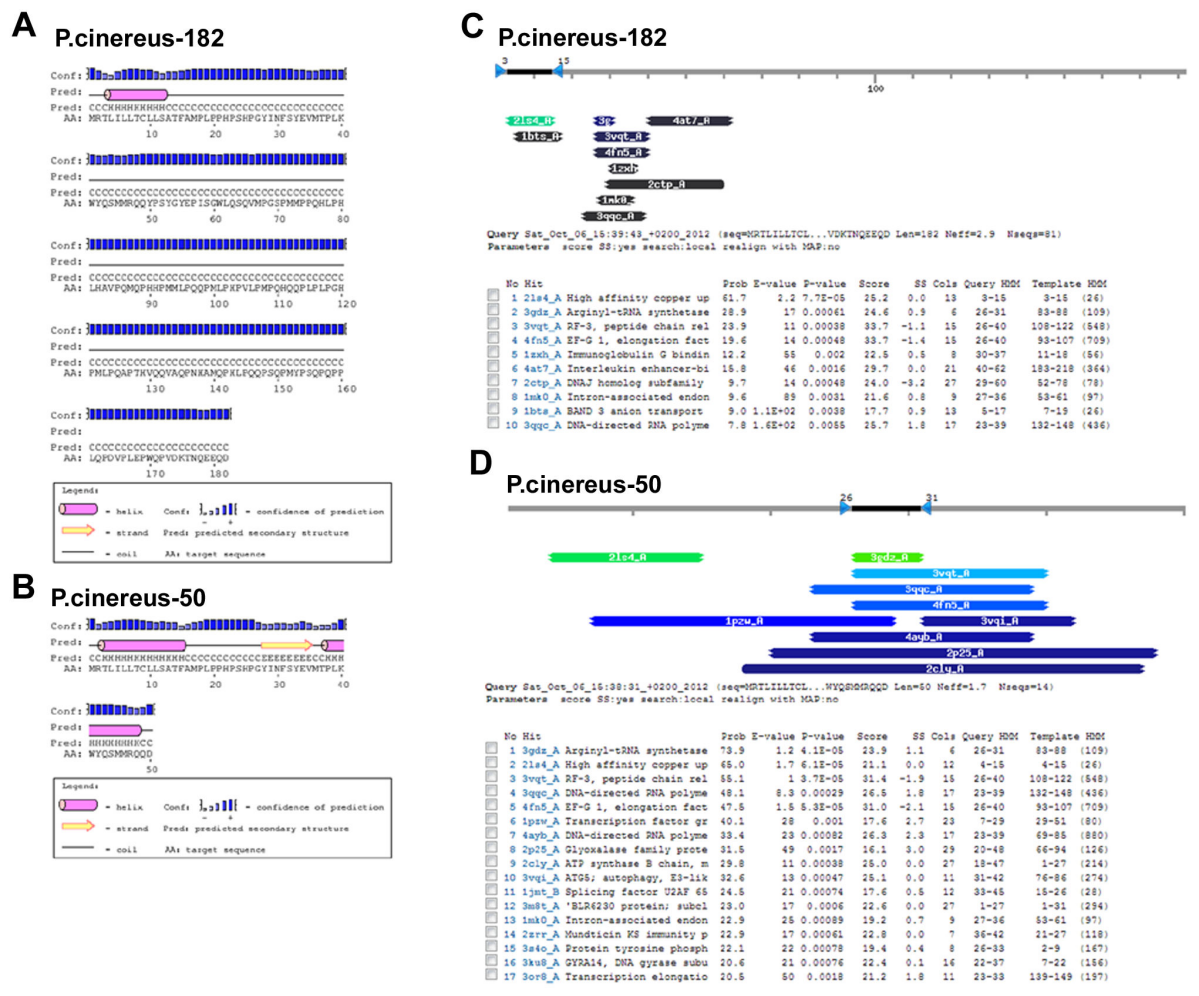
Outsplicing of exon 6 in *P*.*cinereus*-50 has dramatic effects on secondary and tertiary structure. (A–B) **Outsplicing** of exon 6 dramatically affects amelogenin secondary structure of *P*.*cinereus*-50 predicted by Psipred in relation to *P*.*cinereus*-182. One potential helical region was observed in *P*.*cinereus*-182 (AA 4-11), while two potential helical regions were revealed for *P*.*cinereus*-50 (AA 3~15, 38~48) in addition to a beta-strand region (AA 28~35) as revealed by Psipred prediction. (C–D) Exon 6 outsplicing affects the tertiary structure of *P*.*cinereus*-50. *P*.*cinereus*-182 and P.cinereus-50 were used as query sequence for homology detection and structure prediction by HMM-HMM comparison using HHpred. A bar graph summarizes the positions and color-coded significances of the database matches with the probability.

To determine the effect of exon 6 on the amelogenin gene structure, we performed a similar analysis as those of exon 2b. As shown in Figure 6A-B, one potential helix region was revealed in the N-terminal of amelogenin isoform *P*.*cinereus*-182, while two potential helix regions were revealed in the *P*.*cinereus*-50 isoform, one located at the N-terminus (AA 3~15) and the other located at the C-terminus (AA38~48). In addition, there was a strand region (AA 28~35) existed between the two helical regions, indicating that outsplicing of exon 6 dramatically altered amelogenin protein secondary structures.

The effect of exon 6 on the amelogenin tertiary structure in *P*.*cinereus*-50 was performed by employing HHpred for protein homology detection and structure prediction as described previously [20]. As shown in Figure 6C-D, more than half of the protein homologs/domains to *P*.*cinereus*-50 are different from those of N-terminal domains of *P*.*cinereus*-182, suggesting that the exon 6 outsplicing significantly affects the tertiary structure of amphibian amelogenins.

## Discussion

In this study, we selected a caudate amphibian, *Plethodon* *cinereus* , as an animal model and used a gradient PCR to determine potential amelogenin gene splicing forms in salamander tooth organs. Cloning and sequencing the PCR-amplified fragments revealed three isoforms of amelogenin transcripts. One transcript ( *P* *. cinereus* -M) shared the same nucleotide/amino acid sequence as reported in a previous study [22], the other two transcripts ( *P* *. cinereus* -L and *P* *. cinereus* -S) were novel amelogenin splicing forms. The discovery of two novel amelogenin splicing transcripts in salamander suggests that alternative splicing as a mechanism for amelogenin heterogeneity reported in mammalian vertebrates is also employed in amphibians. Previous studies on amphibians [17,19,22], have not reported amelogenin splicing forms. In present study, we have designed primers based on the published amphibian amelogenin sequence and obtained two additional splicing transcript variants in salamander, in which they were not identified in a previous study (Diekwisch et al. 2009). The reason is likely due to the different approach and primers used, where it could be more efficient to detect the alternative splicing transcript variants (Figure 1). As the major enamel matrix protein in the developing tooth organ, amelogenin is considered to be highly conserved [4]. Amelogenin is similar in structure and a full-length amelogenin in mammalian vertebrates consists of 7 exons (1, 2, 3, 4, 5, 6, and 7), in which exon 4 is normally skipped during the processing of amelogenin pre-mRNA. However, later studies discovered two additional exons (exon 8 and exon 9) in rat and mouse tooth organs [11,18], and even an putative exon 4b in mouse, rat, deer mouse and guinea pig genome sequence that separates exon 7 and exon 8 [31,32]. Several laboratories have reported the full-length amelogenin gene in amphibian and sauropsids containing 6 exons (1–3,5–7) in relation to the amelogenin structure in mammals [17,19–22,31]. However, a recent study has identified a novel amelogenin transcript in a reptile that contains 7 exons (1, 2, 3, 5, X, 6, 7) [23], which is not congruent with the findings that the ancestral amelogenin sequence in tetrapods consists of only 6 exons [17,19–22,31,37,38]. The findings that salamander amelogenin transcript contains additional exon 2b in this study is another challenge to current notion about amelogenin gene organization and its evolution. Whether those novel amelogenin exons (exon 2b in *P* *. cinereus* and exon X in *C* *. similis* ) are widely conserved in non-mammalian vertebrate needs to be further explored.

Studies on the alternative splicing of genes have demonstrated that exon size is important for the decision on exon skipping and alternative splicing, in which a small exon is easily skipped and a large exon usually contains internal cryptic splice sites [4]. The amelogenin gene follows that golden rule perfectly, as exon 4 is usually skipped. The exon 2b revealed in this study consists of just 39 nucleotides encoding 13 amino acid residues. As far as we know, it is the smallest exon among amelogenin exons detected so far. Although analysis of amelogenin gene sequence spanning intron 2 in several species did not identify a typical exon/intron boundary, there is indeed a region with a moderate sequence identity to exon 2b nucleotide sequence (39 nucleotides) (not shown). Alignment analysis using exon 2-2b (exon 2 + exon 2b) and exon 2b-3 (exon 2b + exon 3) sequence to search the potential homologous sequence against the *Xenopus* amelogenin genomic sequences showed that exon 2b did not belong to a partial 3’ region of exon 2 or 5’ region of exon 3, suggesting that exon 2b likely originated from intron 2 of the amelogenin gene.

Exon 6 is the largest exon of the amelogenin gene containing several internal cryptic splice sites that could lead to its further sub-division into four domains named exon 6A, exon 6B, exon 6C, and exon 6D [4]. During the processing of amelogenin pre-mRNA, one well-defined amelogenin splicing form (LRAP) contains the majority of N-terminal of exon 6 (exon 6A, exon 6B, exon 6C and few aa of N-terminus of exon 6D) spliced out and played a role in enamel biomineralization and enamel organ epithelial cell differentiation [39,40]. The splicing form of *P*.*cinereus*-50 discovered in present study only contains exons 2, 3, 5 and 7 with exon 6 completely spliced out. As far as we know, this is the first amelogenin splicing transcript in which the entire exon 6 sequence is spliced out. Structure comparison of the putative *P*.*cinereus*-50 with the LRAP splicing form revealed a similar secondary structure in which two potential helix regions existed: one on the C-terminus, the other on the N-terminus, implying the putative *P*.*cinereus*-50 is likely to function in a similar way as LRAP.

Although different approaches have been used to explore the effect of exon 2b on the secondary and tertiary structure of putative *P*.*cinereus*-195, our results did not show a significant effect of exon 2b on *P*.*cinereus*-195 secondary structure; however HHpred prediction identified a few homologs/domains that are different from those of *P*.*cinereus*-182, indicating that exon 2b has an effect on the tertiary structure of putative *P*.*cinereus*-195, thus likely its functions. The novel amelogenin transcripts and the unique exon 2b detected in the salamander will contribute to the understanding of tooth enamel evolution by revealing the conservation and divergence of significant exons of the amelogenin gene throughout vertebrate evolution. Discovery of additional amelogenin sequences will result in enhanced understanding of the origin and evolution of vertebrate teeth.
